# Comparative success of platelet-rich fibrin and mineral trioxide aggregate in direct pulp capping and pulpotomy: a systematic review and meta-analysis

**DOI:** 10.2340/biid.v13.45302

**Published:** 2026-01-21

**Authors:** Malik Alkabazi, Ebtesam Aldieb

**Affiliations:** a Faculty of Dentistry Khalij-Libya, Tripoli, Libya; b Department of Oral Medicine, Oral Pathology, and Oral & Maxillofacial Surgery, University of Tripoli, Tripoli, Libya

**Keywords:** vital pulp therapy, PRF, MTA

## Abstract

**Purpose:**

This systematic review and meta-analysis aimed to compare the clinical success rates of Platelet-Rich Fibrin (PRF) and Mineral Trioxide Aggregate (MTA) when used in pulp-capping and pulpotomy.

**Materials and methods:**

A systematic search of PubMed, Scopus, Embase, and Web of Science was performed. Randomized and non-randomized clinical trials with a minimum 6-month follow-up were included. The risk of bias was assessed using RoB-2 and ROBINS-I tools, and the certainty of evidence was evaluated using GRADE.

**Results:**

Nine studies were included in the qualitative and quantitative synthesis. The meta-analysis at 6 months (7 studies, 436 treated teeth) showed no significant difference between PRF and MTA (odds ratio [OR] 0.72, 95% confidence interval [CI]: 0.29–1.78; P= 0.4799). At 12 months (7 studies, 352 treated teeth), the results also indicated no statistically significant difference (OR 1.50, 95% CI: 0.93–2.43; P= 0.0962), though the point estimate favored PRF.

**Conclusion:**

Within the limitations of this study, PRF and MTA demonstrate broadly similar clinical success rates in pulp-capping and pulpotomy over 6 and 12 months. The low certainty of evidence underscores the need for well-designed, larger randomized controlled trials with extended follow-up to more definitively establish their comparative long-term efficacy.

## Introduction

The term ‘vital pulp therapy’ (VPT) was historically used almost interchangeably with direct pulp-capping (DPC) [1]. However, contemporary definitions have expanded and simplified its scope to encompass all interventions designed to preserve pulp vitality [1]. This broader framework now includes one- and two-step selective caries removal and indirect pulp-capping, aimed at preventing pulp exposure, alongside DPC and pulpotomy procedures [1, 2].

While often discussed as a modern strategy, the concept is far from new, historical records trace its origins back to the 18th century, when Pfaff described the placement of gold over exposed pulp tissue [3, 4]. Over the past two decades, significant developments have reshaped perspectives on VPT, most notably the introduction of bioactive hydraulic calcium silicate cements (HCSCs), which have revitalized interest and progress in this field [5]. These innovations have yielded highly favorable clinical outcomes in pulp-capping and pulpotomy [2], particularly as demonstrated in studies conducted by experienced and well-trained clinicians [6, 7].

Furthermore, recent advances in endodontics have focused on the refinement of regenerative approaches for procedures including pulpotomy, apexogenesis, and bone regeneration following endodontic surgery through the application of platelet concentrates [8–10].

Recent evidence from a multinational survey analysis [11] suggests that clinicians’ approaches to DPC remain diverse and often differ from current guideline recommendations. The survey included 2850 dentists from 16 countries, reported that complete caries removal is still commonly practiced, and calcium silicate-based materials have notably replaced calcium hydroxide-based materials as the preferred capping agents. The same study also indicated that carious pulp exposures frequently lead clinicians to choose more invasive procedures, highlighting a persistent gap between evidence-based guidance and clinical decision-making. These findings emphasize the importance of evaluating bioactive materials such as Platelet-Rich Fibrin (PRF) and Mineral Trioxide Aggregate (MTA) within the context of contemporary endodontic practice.

Several pulp-capping agents have been proposed, including calcium hydroxide Ca (OH)₂, Biodentine, and MTA. However, Ca (OH)₂ presents limitations such as gradual dissolution and inadequate adhesion to dentin [12]. In contrast, MTA is favored over Ca (OH)₂ due to its ability to induce more rapid dentin bridge formation, thereby promoting superior pulp healing. In addition, MTA offers favorable properties including biocompatibility, antibacterial activity, high stability, and excellent sealing capacity [12]. Despite its numerous advantages, several drawbacks have been reported, including high solubility, the presence of toxic components, elevated pH following setting, difficulties in handling, prolonged setting time, higher cost, and the risk of discoloration in both gray and white formulations [13].

Platelet-rich plasma (PRP) has been suggested as a potential pulp-capping material due to its high biocompatibility [14]. Evidence also indicates that PRP facilitates the recruitment and proliferation of pulp mesenchymal stem cells and enhances the mineralization of dental pulp stem cells (DPSC) [15]. PRF is a second-generation platelet concentrate introduced by Choukroun in 2001, incorporates blood-derived elements that support tissue healing [16]. As observed by Ehrenfest, PRF demonstrates several advantages over PRP, including its entirely autologous origin, straightforward preparation, the absence of exogenous agents during processing, and a sustained release of growth factors [17]. The PRF membrane is capable of releasing key growth factors for at least 7 days and up to 28 days, thereby providing a biological scaffold that continuously supports wound healing [17].

In recent years, a growing number of clinical studies have directly compared PRF and MTA as DPC and pulpotomy materials. These studies suggest that both biomaterials hold significant potential in VPT. As only two studies included in the systematic review conducted by Fasoulas [18] and only three studies included in Li [19]. The aim of this systematic review and meta-analysis was to evaluate and synthesize the available clinical evidence comparing PRF and MTA in DPC and pulpotomy and overviewing their relative success rates and the certainty of supporting evidence.

## Materials and methods

### Protocol registration and reporting format

This systematic review and meta-analysis was reported in accordance with the PRISMA [20] (Preferred Reporting Items for Systematic Reviews and Meta-Analyses) guidelines. The methodology was further assessed using AMSTAR 2 tool [21], which evaluates systematic reviews encompassing both randomized and non-randomized healthcare studies, and the protocol was prospectively registered in PROSPERO (CRD420251156455).

### Research question and PICOST framework

The PICOST framework of this review was designed to answer the focused question: What has better success rates PRF or MTA for adults or children with symptomatic or asymptomatic reversible or irreversible pulpitis undergoing DPC and pulpotomy?

**Population (P):** Patients (adults or children) required pulp-capping or pulpotomy.

**Intervention (I):** Platelet rich-fibrin alone or combined with other materials.

**Comparison (C):** Mineral trioxide aggregate.

**Outcome (O):** Success rates of PRF and MTA determined by the absence of spontaneous pain, abnormal responses to thermal tests, tenderness on percussion or palpation, soft tissue changes such as swelling or sinus tract, and radiographic evidences.

**Study designs (S):** Randomized and non-randomized clinical trials.

**Time (T):** Minimum follow-up of 6 months.

### Eligibility criteria

#### Inclusion criteria

1. Primary and permanent teeth treated with PRF or MTA for pulp-capping or pulpotomy.
2. Minimum follow-up of 6 months.
3. Clinical trials.
4. No restriction regarding the language and geographic area.

#### Exclusion criteria

1. Studies did not compare PRF with MTA or did not clearly report the success rate.
2. Study designs other than clinical trials (Case reports, case series, observational and in vivo or in vitro studies).

### Search strategy

A systematic search was carried out in four electronic databases; PubMed, Scopus, Embase, and Web of Science. The search was based on MeSH terms and free-text keywords as following: ((‘Platelet-Rich Fibrin’[Mesh]) OR (PRF)) AND ((MTA) OR (‘Mineral trioxide aggregate’) OR (biodentine) OR (Calcium hydroxide)) AND ((‘Endodontics’[Mesh]) OR (‘Pulp capping’) OR (‘Pulp Capping and Pulpectomy Agents’[Mesh])). The full search strategy presented in Table 1.

**Table 1:** The full search strategy.

Database	Search query	*N*
**PubMed:** no filters applied, date: August 27, 2025		
((‘Platelet-Rich Fibrin’[Mesh]) OR (PRF)) AND ((MTA) OR (‘Mineral trioxide aggregate’) OR (biodentine) OR (Calcium hydroxide)) AND ((‘Endodontics’[Mesh]) OR (‘Pulp capping’) OR (‘Pulp Capping and Pulpectomy Agents’[Mesh]))		36
**Scopus** Filter: Article date: August 27, 2025	((“Platelet-Rich Fibrin”) OR (PRF)) AND ((MTA) OR (“Mineral trioxide aggregate”) OR (biodentine) OR (Calcium hydroxide)) AND ((“Pulp capping”) OR ( “Pulp Capping and Pulpectomy Agents” ) ) AND ( LIMIT-TO ( DOCTYPE ,”ar” ))	191
**Embase** Filter: No filter date: August 27, 2025	(‘platelet-rich fibrin’/exp OR ‘platelet-rich fibrin’ OR prf) AND (‘mta’/exp OR mta OR ‘mineral trioxide aggregate’/exp OR ‘mineral trioxide aggregate’ OR ‘biodentine’/exp OR biodentine OR ‘calcium hydroxide’/exp OR ‘calcium hydroxide’ OR ((‘calcium’/exp OR calcium) AND (‘hydroxide’/exp OR hydroxide))) AND (‘pulp capping’/exp OR ‘pulp capping’ OR ‘pulp capping and pulpectomy agents’/exp OR ‘pulp capping and pulpectomy agents’)	126
**Web Of Science** Filter: No filter date: August 27, 2025	(‘platelet-rich fibrin’/exp OR ‘platelet-rich fibrin’ OR prf) AND (‘mta’/exp OR mta OR ‘mineral trioxide aggregate’/exp OR ‘mineral trioxide aggregate’ OR ‘biodentine’/exp OR biodentine OR ‘calcium hydroxide’/exp OR ‘calcium hydroxide’ OR ((‘calcium’/exp OR calcium) AND (‘hydroxide’/exp OR hydroxide))) AND (‘pulp capping’/exp OR ‘pulp capping’ OR ‘pulp capping and pulpectomy agents’/exp OR ‘pulp capping and pulpectomy agents’)	26

### Article selection and data extraction

All records obtained from the electronic database searches were systematically imported into EndNote reference management software (Version 20.0.1, Clarivate Analytics). To ensure accuracy and avoid redundancy, duplicate citations were first identified and removed through the software’s automated duplicate-detection tool, after which an additional manual verification was performed to confirm the precision of the process. The resulting deduplicated dataset was then transferred into Microsoft Excel, which served as the primary platform for organizing references and facilitating the subsequent screening stages.

The screening process was carried out in two distinct phases. Initially, titles and abstracts of the retrieved records were independently evaluated by two reviewers (M.A. and E.A.) to determine their relevance according to the predefined eligibility criteria. Studies deemed potentially eligible at this stage were advanced to full-text assessment, which was again performed independently by the same reviewers. In cases where disagreements or uncertainties arose during either stage, resolution was achieved through discussion, thereby ensuring methodological consistency and minimizing potential bias.

For each study that satisfied the inclusion criteria, a structured and comprehensive data extraction process was undertaken. Extracted information encompassed all relevant study characteristics, methodological details, interventions, outcomes, and key findings whenever reported. This approach ensured that the dataset was both robust and sufficiently detailed to support subsequent qualitative synthesis and quantitative analysis.

### Qualitative assessment and certainty of evidence

The methodological quality of included studies was evaluated by (M.A. and E.A.) using established tools appropriate to their design. Randomized controlled trials were assessed with the Cochrane Risk of Bias 2.0 (RoB-2) tool [22], which examines five domains: randomization and allocation concealment, deviations from intended interventions, completeness of outcome data, reliability of outcome measurements, and selective reporting, with judgments categorized as low risk, some concerns, or high risk. Non-randomized studies were appraised with the ROBINS-I tool [23], which evaluates seven domains including confounding, participant selection, intervention classification, deviations from intended interventions, missing data, outcome measurement, and selective reporting, rated as low, moderate, serious, or critical risk. Overall assessments were summarized and visualized using Robvis [24], ensuring a transparent and comprehensive appraisal of study validity across both randomized and observational evidence. In addition, the certainty of evidence for each outcome was graded by two reviewers (M.A. and E.A.) using the GRADE (Grading of Recommendations, Assessment, Development, and Evaluations) approach, any discrepancies were resolved through discussion. A summary of findings was generated in tabular form through the GRADEpro GDT software (McMaster University and Evidence 9 Prime, 2025. gradepro.org) to provide a transparent overview of the evidence quality across outcomes.

### Data analysis

The characteristics of all eligible studies were systematically organized into summary tables and described using descriptive statistics to provide a comprehensive overview of study design, participants’ demographics, interventions, and measured outcomes. All statistical analyses were performed in R (version 4.4.3; RStudio, Vienna, Austria) using the ‘meta’ package. To account for anticipated clinical and methodological variability across studies, pooled effect estimates were generated under a random-effects model. Results were expressed as odds ratios (ORs) with corresponding 95% confidence intervals (CIs). Statistical heterogeneity was assessed using the I² statistic, with thresholds interpreted as low (<50%), moderate (50–75%), and substantial (>75%) heterogeneity. A *p*-value <0.05 was regarded as the threshold for statistical significance. In addition, subgroup analyses were carried out to compare outcomes between studies investigating pulp-capping and those evaluating pulpotomy, allowing for exploration of potential differences in clinical performance.

## Results

### Literature search

**Figure 1:**
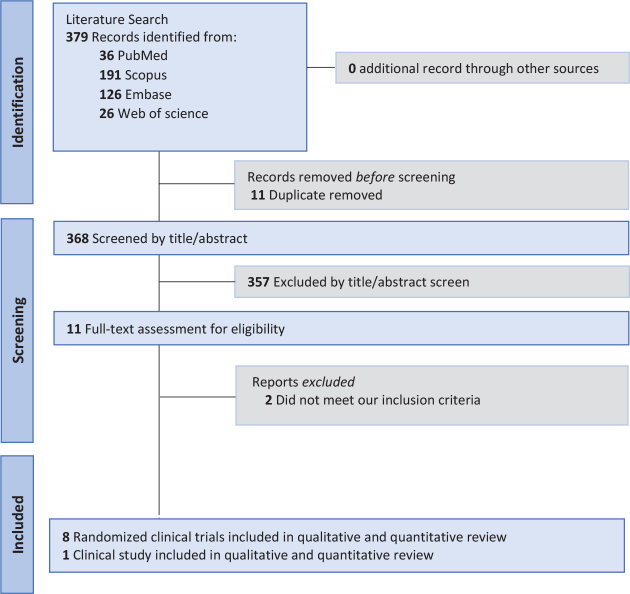
PRISMA flowchart.

The comprehensive electronic database search initially yielded 379 records. After duplicate removal, a total of 368 unique citations were available for screening. Titles and abstracts of these records were systematically screened to determine their relevance to the research question. At this stage, articles that were clearly unrelated to the objectives of the review were excluded. Records that appeared potentially relevant, or for which eligibility could not be confidently determined from the abstract alone, were advanced to full-text review. A total of 11 full-text articles were retrieved and subjected to detailed assessment against the predefined inclusion and exclusion criteria. This rigorous process ensured that only studies with appropriate design, population, intervention characteristics, and outcome measures were retained. The full-text evaluation stage was carried out with particular attention to methodological quality and alignment with the objectives of the review, ensuring transparency, reproducibility, and consistency in study selection. Ultimately, nine studies [25–33] were included in the qualitative and quantitative review (Figure 1).

### Characteristics of the included studies

All of the included studies were RCTs except one study [33] was non-RCT, published between 2014 and 2025. The studies compared PRF against MTA, Calcium Hydroxide, Nano-Hydroxyapatite, and PRP. One study [30] evaluated combinations such as PRF + MTA or PRF + Ca (OH)₂.

Both pulp-capping [25–27, 33] and pulpotomy [28–32] procedures were assessed. Pulp-capping studies mainly involved young permanent teeth or adult teeth with pulpitis, while pulpotomy studies focused on primary teeth or permanent teeth with incomplete root formation.

Sex distribution was reported inconsistently; in studies that reported it, male-to-female ratios varied and some of them did not report the ratio. Age ranges spanned from early childhood (5 years) in primary tooth studies to adults up to 45 years.

Furthermore, conclusions across all studies revealed that PRF showed comparable success rates to MTA, with several studies suggesting PRF as a promising or acceptable alternative for VPT. One study [28] highlighted unique benefits, such as reduced pulp canal obliteration, while others emphasized that differences among tested materials (PRF, MTA, Ca (OH)₂) were not statistically significant (Tables 2 and 3).

**Table 2:** Characteristics of the included studies.

Study	Study design	Country	Groups	Total treated teeth	Procedure	M/F	Age range	Conclusion
Abuhashema, 2025	RCT	Egypt	PRF and MTA	108	Pulp-capping	43/65	15–40 years	‘DPC with the novel pulp-capping agent, PRF, exhibits a clinical and radiographic success rate comparable to that of MTA’.
Tiwari, 2024	RCT	India	PRF and MTA	50	Pulp-capping	35/15	5–9 years	‘PRF can be used successfully as an appropriate alternative material in DPC of primary teeth when compared with MTA’.
Eid, 2022	RCT	Syria	PRF, MTA, and Nano-Hydroxyapatite	60	Pulpotomy	30/30	6–12 years	‘PRF showed a statistically lower tendency to pulp canal obliteration when compared to the MM-MTA (auto-mixed) and NHA group (manually mixed) (*p* < 0.05)’.
Shobana, 2022	RCT	India	PRF, MTA, and PRP	27	Pulp-capping	N/A	18–45 years	‘PRF could be the future of vital pulp therapy for adult permanent teeth with reversible pulpitis’.
Singh, 2020	Clinical trial	India	PRF, MTA, and Ca (OH)_2_	60	Pulp- capping	N/A	N/A	‘Pulp-capping agents such as Ca (OH)2, MTA, and PRF yielded similar success rate when used in teeth with irreversible pulpitis’.
Manhas, 2019	RCT	India	PRF + MTA, PRF + Ca (OH)_2_, and MTA	180	Pulpotomy	N/A	6–9 years	‘All materials used in the present study are equally effective as the success rates of all the materials are statistically insignificant’.
Patidar, 2017	RCT	India	PRF and MTA	50	Pulpotomy	N/A	5–9 years	‘PRF group could suggest it as an acceptable alternative in pulpotomy of primary teeth. PRF holds a promising future in the area of primary tooth vital pulp therapy’.
Kumar, 2016	RCT	India	PRF, MTA, and Ca (OH)_2_	41	Pulpotomy	24/18	14–32 years	‘There exists no significant difference between the success rates of Ca (OH)_2_, MTA, and PRF as pulpotomy agents in teeth with irreversible pulpitis’.
Keswani, 2014	RCT	India	PRF and MTA	112	Pulpotomy	N/A	6–12	‘PRF can be used as a pulpotomy agent in permanent teeth with incomplete root development’.

Abbreviations: RCT, randomized controlled trial; PRF, Platelet Rich-Fibrin; MTA, Mineral Trioxide Aggregate; Ca (OH)2, Calcium hydroxide; PRP, Platelet Rich-Fibrin; DPC, Direct Pulp-Capping.

**Table 3:** An overview of the included studies.

Study	Pulpotomy	Type of teeth included	Root maturation	Pulpal diagnosis	Biomaterial		Follow-up (Months)
					MTA	PRF	
Abuhashema, 2025	-	Permanent posterior teeth	Mature	Reversible pulpitis	MTA (Angelus, Brazil) was prepared according to the manufacturer’s instructions.	PRF was prepared following Choukroun’s protocol by collecting 5 mL of venous blood in plain tubes without anticoagulant and centrifuging at 3000 rpm for 10 minutes (≈1007 × g) using a tabletop centrifuge (800D, Biofield Medical, China; rotor radius ≈10 cm).	6 and 12.
Tiwari, 2024	-	Primary molars	N/A	Unspecified	The pulp stump was covered with fast-setting MTA (Angelus). The material was prepared by mixing MTA powder with sterile water at a 3:1 ratio, as per the manufacturer’s instructions.	PRF was prepared following Choukroun’s protocol, with venous blood collected in 10 mL tubes and centrifuged at 2700 rpm for 12 minutes.	1, 3, 6, 9, and 12
Eid, 2022	N/A	Permanent molars	Immature	Unspecified	After hemostasis, MM-MTA auto-mixed CSBC ‘Bioceramics’ (G1) was applied directly to the pulp stumps and lightly condensed with an amalgam plugger. Sterile paper points were used to achieve a 2–3 mm coronal thickness.	PRF was prepared using Choukroun’s protocol, in which 5 mL of venous blood was collected in plain 10 mL tubes and centrifuged immediately at 3000 rpm for 10 minutes (Hettich, Hohberg, Germany).	6 and 12
Shobana, 2022	-	Permanent posterior teeth	Mature	Reversible pulpitis	MTA was mixed with the manufacturer-supplied liquid on a sterile glass slab using a stainless-steel spatula and placed over the pulp exposure to a thickness of 2 mm. The cavity floor was then gently dabbed with a moist sterile cotton pellet.	PRF was prepared in accordance with Choukroun’s protocol.	3, 6 and 12
Singh, 2020	-	Permanent molars	N/A	Irreversible pulpitis	N/A	N/A	6 and 24
Manhas, 2019	FP	Primary teeth	N/A	Unspecified	MTA was placed in the pulp chamber, followed by a thick layer of glass ionomer cement (GIC).	PRF was prepared following Choukroun’s protocol by collecting 5 mL of venous blood in plain 10 mL tubes and centrifuging immediately at 2400–2700 rpm for 12 minutes.	1, 3, and 6
Patidar, 2017	N/A	Primary molars	N/A	Unspecified	A paste was prepared by mixing MTA powder with sterile water at a 3:1 ratio, as per the manufacturer’s instructions.	Samples were prepared using Choukroun’s procedure by collecting venous blood in 10 mL tubes without anticoagulant and centrifuging immediately at 2700 rpm for 12 minutes.	1, 3, and 6
Kumar, 2016	FP	Permanent molars	Mature	Irreversible pulpitis	ProRoot MTA powder (Dentsply Maillefer, Ballaigues, Switzerland) was mixed with distilled water on a sterile glass slab and applied as a ~2 mm layer over the exposed pulp.	PRF was prepared according to Choukroun’s technique, involving immediate centrifugation of venous blood without anticoagulant.	6 and 12
Keswani, 2014	N/A	Permanent molars	Immature	Unspecified	One gram of cement powder was combined with 0.3 mL of distilled water (3:1 ratio) according to the manufacturer’s guidelines. Mixing for 30 seconds produced a putty-like consistency with a working time of about 5 minutes.	PRF was prepared as described by Choukroun . Five milliliters of blood were drawn into a 10 mL anticoagulant-free tube and immediately centrifuged at 3000 rpm for 10 minutes using a tabletop centrifuge (REMI Laboratories, Mumbai, India).	6, 12, and 24

Abbreviations: PRF, Platelet Rich-Fibrin; MTA, Mineral Trioxide Aggregate; FP, Full Pulpotomy; MM-MTA, Fast setting MTA.

### Qualitative assessment

The risk of bias among randomized controlled trials varied across domains. Most studies demonstrated a low risk in several domains; however, some concerns were observed, particularly in relation to deviations from intended interventions and measurement of outcomes. Three trials [26, 27, 30] were judged at overall high risk of bias, whereas the remaining trials ranged from low to some concerns (Figure 2A–B).

**Figure 2:**
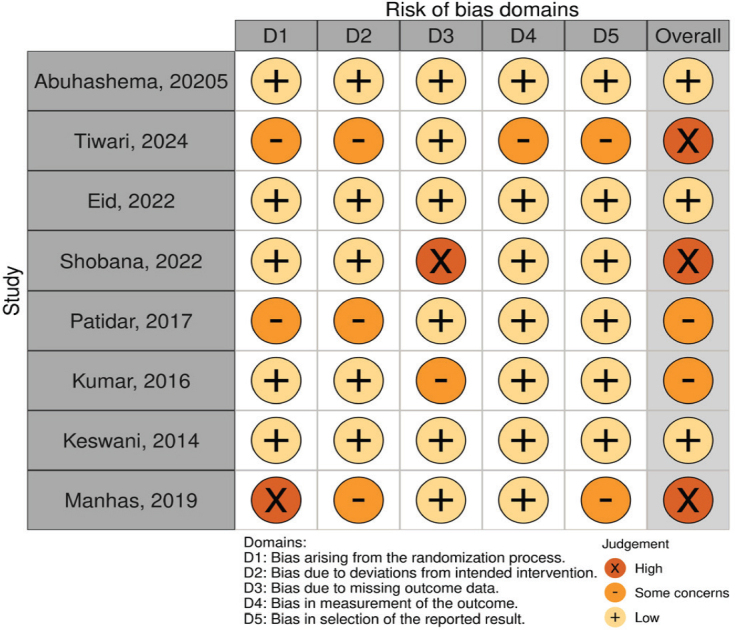
(A) Risk of bias (RoB-2) judgement.

**Figure 2:**
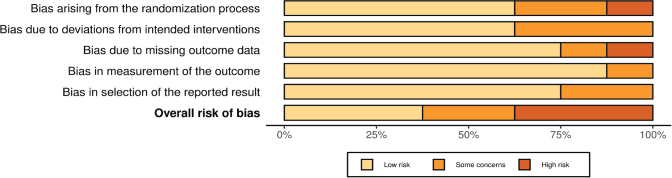
(B) Summary of risk of bias (RoB-2) judgment.

For the single non-randomized study [33] assessed using ROBINS-I (Singh, 2020), the overall risk of bias was judged as critical, primarily due to serious concerns regarding confounding, missing data, and selective reporting, despite low risk in other domains (Figure 2C–D).

**Figure 2:**
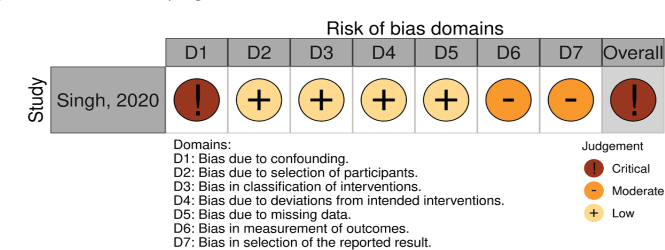
(C) Risk of bias (ROBINS-1) judgement.

**Figure 2:**
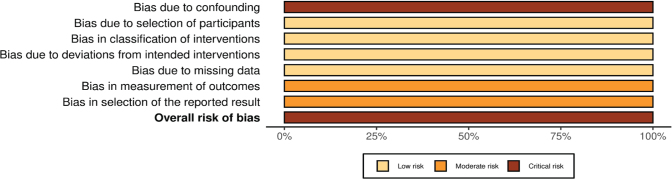
(D) Summary of risk of bias (ROBINS-1) judgment.

### Certainty of evidence

The overall certainty of the evidence for the success rates of PRF compared to MTA was assessed as low at both 6 and 12 months, according to the GRADE criteria. This rating was due to most studies in the analysis having results that crossed the line of no effect (null) and the inclusion of studies with a high risk of bias. A summary of findings are presented in Table 4.

**Table 4:** A summary of findings (PRF compared with MTA for pulp-capping and pulpotomy).

Outcomes	№. of participants (studies)	Certainty of the evidence (GRADE)	Relative effect (95% CI)	Anticipated absolute effects Assumed success	
				Success with MTA	Success difference with PRF
Success rate at 6 months	436 (6 RCTs and 1 non-RCT)	⨁⨁◯◯ Low^a^	**OR 0.72** (0.29–1.78)	85 per 100	5 fewer per 100 (23 fewer to 6 more)
Success rate at 12 months	392 (6 RCTs and 1 non-RCT)	⨁⨁◯◯ Low^a,b^	**OR 1.50** (0.93–2.43)	63 per 100	9 more per 100 (2 fewer to 18 more)

*The risk in the intervention group (and its 95% confidence interval) is based on the assumed risk in the comparison group and the relative effect of the intervention (and its 95% CI).

Abbreviations: PRF, Platelet Rich-Fibrin; MTA, Mineral Trioxide Aggregate; CI: confidence interval; OR: odds ratio.

**GRADE Working Group grades of evidence.**

**High certainty:** we are very confident that the true effect lies close to that of the estimate of the effect.

**Moderate certainty:** we are moderately confident in the effect estimate: the true effect is likely to be close to the estimate of the effect, but there is a possibility that it is substantially different.

**Low certainty:** our confidence in the effect estimate is limited: the true effect may be substantially different from the estimate of the effect.

**Very low certainty:** we have very little confidence in the effect estimate: the true effect is likely to be substantially different from the estimate of effect.

Explanations.

a Most of the studies cross the null.

b High risk of bias studies.

### Meta-analysis

#### Success rate at 6 months

**Figure 3:**
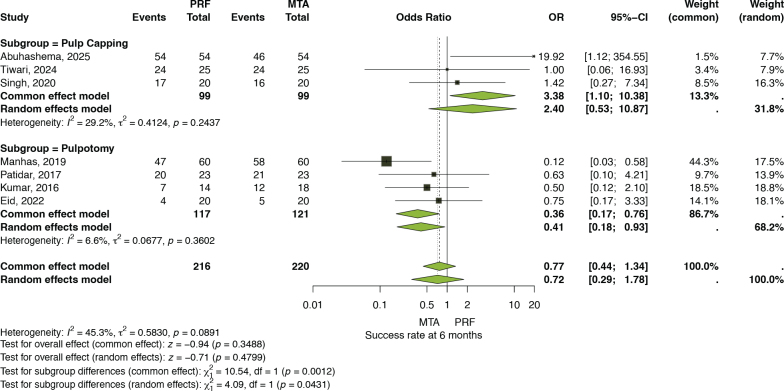
Success rate at 6 months (Subgroup analysis based on procedure).

At the 6-month follow-up, data from seven clinical trials [25, 26, 28–31, 33] involving 436 cases were pooled. The overall analysis showed no significant difference between PRF and MTA. Using the common-effect model, the OR was 0.77 (95% CI: 0.44–1.34, *p* = 0.3488), while the random-effects model yielded an OR of 0.72 (95% CI: 0.29–1.78, *p* = 0.4799). Heterogeneity was moderate (*I*² = 45.3%, *τ*² = 0.5830, *p* = 0.0891). Subgroup analyses revealed contrasting findings; the common-effect model indicated a statistically significant difference between pulp-capping and pulpotomy procedures (df = 1, *p* = 0.0012), whereas the random-effects model revealed (df = 1, *p* = 0.0431) (Figure 3).

#### Success rate at 12 months

**Figure 4:**
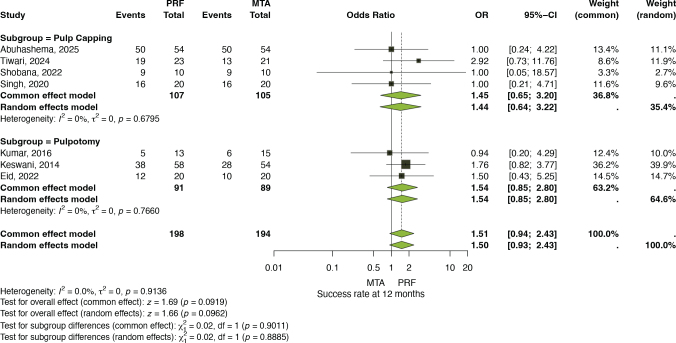
Success rate at 12 months (Subgroup analysis based on procedure).

At the 12-month follow-up, seven trials [25–29, 32, 33] with a total of 392 cases were analyzed. Both models demonstrated comparable results, with the common-effect model yielding an OR of 1.51 (95% CI: 0.94–2.43, *p* = 0.0919) and the random-effects model producing an OR of 1.50 (95% CI: 0.93–2.43, *p* = 0.962). Heterogeneity was negligible (*I*² = 0.0%, *τ*² = 0, *p* = 0.9136), indicating consistent outcomes across studies. Subgroup analyses revealed no evidence of difference between pulp-capping and pulpotomy (*p* > 0.8 for both models). Although the pooled estimates were not statistically significant, the direction of effect consistently suggested slightly higher success rates with PRF compared to MTA (Figure 4).

**Figure 5:**
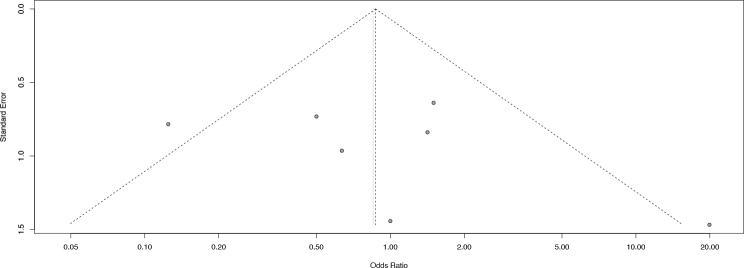
Funnel plot of 6 months.

**Figure 6:**
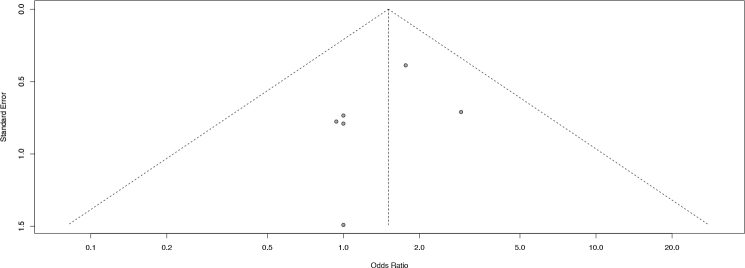
Funnel plot of 12 months.

Furthermore, visual inspection of the funnel plots at 6 and 12 months follow-up did not reveal substantial asymmetry, suggesting a low likelihood of publication bias. However, since less than 10 studies were included in each analysis, Egger’s test was not conducted, in accordance with recommended methodological standards (Figures 5 and 6).

## Discussion

The present systematic review and meta-analysis results across both follow-up (6 and 12 months) periods indicate that PRF and MTA achieve broadly similar clinical outcomes. At 6 months, heterogeneity and subgroup inconsistencies limit the certainty of the pooled estimates, while at 12 months the evidence is likely more stable and homogeneous, with a persistent but non-statistically significant favoring PRF.

### Results in relation to previous evidence

Previous pairwise and network meta-analyses have consistently shown that calcium silicate materials, especially MTA, achieve better outcomes than calcium hydroxide for DPC and pulpotomy [18, 34, 35]. However, a meta-analysis published in 2023 directly compared PRF with MTA [18], and this was based on just two studies. Similarly, the findings of the present meta-analysis revealed an overall 28% higher but not significant odds of treatment success within the first 6 months of follow-up. Interestingly, when PRF was specifically used as a pulp-capping material, the odds of success appeared higher, with an increase of approximately 140%; however, the difference was not statistically significant. Although this observation points toward a potentially strong clinical benefit, caution is warranted in interpreting these results. ORs, particularly in the context of a common-effect model, may exaggerate the apparent magnitude of treatment effects, especially when event rates are high, as shown in Figure 3. Therefore, while the data suggest that PRF could offer a meaningful advantage over MTA, these results should be considered exploratory and interpreted in light of the methodological limitations and the variability among the included studies.

Our pooled 12-month estimate showed an OR of 1.50 (95% CI 0.93–2.43) favoring PRF over MTA, but this effect did not reach statistical significance and between-study heterogeneity was negligible (*I*² = 0%). Thus, the present synthesis finds no conclusive difference between PRF and MTA for success at 12 months, although the point estimate suggests a modest benefit with PRF.

This finding aligns with the several recent randomized clinical trials that reported broadly comparable short-term clinical and radiographic outcomes for PRF and MTA when used as DPC or pulpotomy agents. For example, a randomized trial in primary molars found similar 12-month clinical and radiographic success between PRF and MTA [26]. A larger, double-blinded randomized study of traumatically exposed permanent teeth likewise reported comparable clinical success and evidence of reparative dentin formation between the two materials at 1 year [25].

Interpreting these comparative results requires placement within the broader evidence base for VPT materials. Systematic reviews and meta-analyses consistently show that MTA and other calcium silicate cements outperform traditional calcium hydroxide for DPC and pulpotomy, and are broadly equivalent to other modern bioactive cements such as Biodentine in many settings [18].

Mechanistically, these clinical similarities are understandable. MTA (HCSC) promotes hard-tissue formation through sustained calcium ion release, a localized rise in pH together with the development of a mineralized interface contributes to effective sealing and promotes dentin formation [36]. By contrast, PRF is an autologous second generation platelet concentrates fibrin scaffold that concentrates platelets, leukocytes and growth factors, thereby promoting angiogenesis, cell recruitment, soft-tissue healing, and biological processes [37, 38] that could support pulp survival and reparative dentinogenesis in exposed pulps. Furthermore, Huang [39] and Zhang [40] revealed that PRF enhanced healing of the pulp and had no cytotoxic effects on stem cells of the pulp. These complementary biological modes of action help explain why both materials can produce favorable clinical outcomes in the short term [41].

### Strength and limitations

This study provides a concise yet comprehensive overview of the direct comparison between PRF and MTA in pulp-capping and pulpotomy procedures. Several practical and methodological factors limit definitive conclusions. Firstly, PRF protocols vary substantially (centrifugation speed/g-force, time, tube type and whether A-PRF/i-PRF modifications are used), and these parameters materially alter cell and growth factor content [42]; such variability reduces comparability across trials. Secondly, MTA products differ in handling, setting time and potential for discoloration; trial heterogeneity in the exact MTA formulation and application technique is common. Thirdly, many trials remain relatively small, with follow-up limited to 12 months in several studies – a follow-up duration that may not capture late failures or differences in long-term dentin bridge quality. Finally, the treated teeth across studies are not limited to a specific clinical situation, as the included populations encompass both mature and immature teeth, cases of reversible and irreversible pulpitis, and patients ranging from children to adults – all of which contribute to further clinical heterogeneity.

### Implications for future research

Current randomized evidence, along with available meta-analyses, suggests that PRF and MTA demonstrate broadly comparable short-term success in VPT. Nevertheless, the evidence base remains limited and heterogeneous, particularly when contrasted with the more robust and consistent data supporting MTA over older materials such as calcium hydroxide. To build more definitive conclusions and enable reliable pairwise and network meta-analyses, future randomized trials should address several key methodological issues. These include the pre-specification and standardization of PRF preparation protocols with explicit reporting of centrifugation force (RCF/g) and duration, the use of a single, well-defined MTA formulation to minimize material-related variability, and blinded outcome assessment wherever feasible to reduce bias. Moreover, extended follow-up of at least 24 months, combined with the adoption of standardized clinical and radiographic outcome measures, is critical to ascertain true long-term effectiveness. Only through such rigorously designed trials can the comparative effectiveness of PRF and MTA be meaningfully established and incorporated into future network meta-analyses.

## Conclusion

Within the limitations of this study, MTA demonstrated a statistically significant advantage over PRF for pulpotomy at 6 months. However, overall effects at both 6 and 12 months did not reveal a definitive difference between the two biomaterials in either direct pulp capping or pulpotomy outcomes. Well-designed, larger randomized controlled trials with extended follow-up periods are needed to more conclusively determine the long-term comparative efficacy of MTA and PRF.
